# Comparing the quality of uniportal and multiportal video-assisted anatomical resection for primary lung cancer: the preliminary results of a single centre audit

**DOI:** 10.1186/1749-8090-10-S1-A235

**Published:** 2015-12-16

**Authors:** Veena Surendrakumar, Paul R Vaughan, Jagan Rao, John G Edwards, Laura Socci

**Affiliations:** 1 Department of Cardiothoracic Surgery, Northern General Hospital, Sheffield, UK

## Background/Introduction

Clear resection margins and appropriate lymph node dissection are fundamental aspects of complete lung cancer resection, as defined by the International Association for the Study of Lung Cancer (IASLC). However, no reports to date have compared these outcomes in uniportal versus multiportal approaches to video-assisted thoracoscopic surgery (VATS).

## Aims/Objectives

We present a large case series comparing the quality of uniportal and multiportal VATS lung resection for lung cancer.

## Method

A retrospective review was performed on all patients who underwent VATS anatomical resection for primary lung malignancy between January 2013 and April 2015. Data collection comprised patient demographics, operative technique and pathological staging. Primary outcomes were presence of clear resection margins and quality of lymph node dissection in accordance with IASLC guidelines. Statistical differences between uniportal and multiportal approaches were calculated using the χ2 and student's t-test.

## Results

A total of 182 patients were included in the study, 101 in the uniportal group and 81 in the multiportal group. Multiportal approaches comprised of two-port access in 61 patients and three-port access in 20 patients. Lobectomies were performed in 163 patients and anatomical segmentectomy performed in the remainder.

There were no significant differences in involvement of bronchial, mediastinal or vascular resection margins with 95% of tumours reported as fully excised in both groups. The mean number of nodal stations dissected was 4.14 using uniportal access and 3.97 via the multiportal approach (p = 0.326). The difference in mean number of mediastinal stations dissected between both groups was also not significant (mean 2.05, uniportal; mean 2.15, multiportal; p = 0.402). Almost all resections involved hilar or intrapulmonary nodal sampling (99% in both groups).

## Discussion/Conclusion

Quality of resection in single port access for VATS anatomical lung resection is comparable to the traditional multiportal approach in our single centre series. Greater awareness of the importance of lobe-specific nodal dissection may improve operator outcomes.

